# Podocalyxin-like protein expression in primary colorectal cancer and synchronous lymph node metastases

**DOI:** 10.1186/1746-1596-8-109

**Published:** 2013-07-02

**Authors:** Anna H Larsson, Björn Nodin, Ingvar Syk, Ingrid Palmquist, Mathias Uhlén, Jakob Eberhard, Karin Jirström

**Affiliations:** 1 Department of Clinical Sciences, Division of Pathology, Lund University, Skåne University Hospital, SE-221 85 Lund, Sweden; 2 Department of Clinical Sciences, Division of Oncology, Lund University, Skåne University Hospital, 221 85 Lund, Sweden; 3 Department of Clinical Sciences, Division of Surgery, Lund University, Skåne University Hospital, 205 02 Malmö, Sweden; 4 Science for Life Laboratory, AlbaNova University Center, Royal Institute of Technology, 106 91 Stockholm, Sweden; 5 School of Biotechnology, AlbaNova University Center, Royal Institute of Technology, 106 91 Stockholm, Sweden

## Abstract

**Aims:**

Previous studies have shown that membranous expression of podocalyxin-like protein (PODXL) is associated with poor prognosis in colorectal cancer (CRC). In this study, we compared PODXL expression in primary CRC and synchronous lymph node metastases. We further analyzed whether its expression changed in rectal tumours after neoadjuvant radiation therapy.

**Methods and results:**

The studied cohort consists of 73 consecutive patients from the South-Swedish Colorectal Cancer Biobank. Immunohistochemical PODXL expression was examined on full-face sections from all primary tumours and all 140 available lymph node metastases from 31 cases. Membranous PODXL expression was denoted in 18/73 (24,7%) primary tumours, with a high concordance between primary and metastatic lesions. While all negative primary tumours had negative metastases, some PODXL positive primaries had a varying proportion of positive and negative metastatic lymph nodes. PODXL expression was also found to be mainly unaltered in pre- and post-irradiation surgically resected tumour specimens in rectal cancer patients (n=16).

**Conclusions:**

The findings in this study suggest that analysis of PODXL expression in the primary tumour is sufficient for its use as a prognostic and treatment predictive biomarker in CRC, also in patients with metastatic disease.

**Virtual slides:**

The virtual slide(s) for this article can be found here: http://www.diagnosticpathology.diagnomx.eu/vs/9014177329634352

## Introduction

Every year more than 1,2 million people worldwide are diagnosed with CRC and although CRC mortality is progressively declining, it still remains the second most common cause of cancer death in the Western world. Prognosis is mostly dependent on disease stage at diagnosis, however, outcome may vary considerably even within the same tumour stage. Thus, there is a great need for additional prognostic biomarkers to better identify patients with a high risk of developing metastases.

Podocalyxin-like protein (PODXL) is a transmembrane glycoprotein with anti-adhesive properties, first identified in the kidney where it plays a vital role in maintaining filtration pathways [1]. PODXL is also expressed by vascular endothelial cells [1], platelets [2], and hematopoietic stem cells [3]. The role of PODXL in cancer was first described in testicular cancer [4]. Since then, PODXL has been found to be overexpressed in numerous cancer types and associated with a more aggressive tumour phenotype and poor outcome in breast [5], prostate [6], colorectal [7,8] ovarian [9] and bladder cancer [10]. The poor prognosis seems to be conferred by PODXL expression on the membrane of tumour cells, and predominantly at the invasive tumour front [7,11], further indicating an integral role for this protein in the progression of some tumours.

Our previous studies have shown that PODXL is an independent predictor of poor prognosis in CRC and a possible future tool for selecting high risk patients for adjuvant treatment [7]. Given the potential clinical utility of PODXL, we conducted the present study to investigate the grade of concordance in terms of PODXL expression between primary colorectal tumours and corresponding lymph node metastases, and also the effect of neoadjuvant radiation therapy on PODXL expression in rectal cancer. Moreover, since previous studies were retrospective and based on analysis of tissue-microarrays (TMAs), a secondary objective was to examine whether analysis of full-face sections reveals a larger proportion of tumours with membranous PODXL expression.

## Materials and methods

### Patients

The study cohort included all patients in the prospective South-Swedish Colorectal Cancer Biobank (STABB) who were surgically treated for primary CRC at Skåne University Hospital in Malmö, Sweden between January 1^st^ and September 30^th^ 2012 (n=74). One patient with complete histopathological response, i.e. abscence of tumour cells in the surgical specimen post-irradiation, was excluded from the study. Thirty-two (43,8%) of the remaining 73 patients had lymph node metastases and four (5,5%) had stage IV disease with liver metastases. Median age at diagnosis was 72 years (range 44–92 years).

Twenty-one patients with rectal cancer received neoadjuvant radiation treatment. Eighteen of these patients were given 25 Gy and three patients received a long radiation therapy of 50,4 Gy combined with per oral capecitabine prior to surgery.

Histopathological, clinical and treatment data were obtained from pathology and hospital records. Patient and tumour characteristics are summarized in Table 1.

**Table 1:** PODXL expression and clinicopathological parameters of the cohort

		**PODXL neg**	**PODXL pos**	
n (%)	73 (100)	55 (75,3)	18 (24,7)	p-value
**Age**				
≤75	49 (67,1)	36 (65,5)	13 (72,2)	0.77
>75	24 (32,9)	19 (34,5)	5 (27,8)	
**Gender**				
Female	36 (49,3)	20 (36,4)	16 (88,9)	<0,001
Male	37 (50,7)	35 (63,6)	2 (11,1)	
**T**				
1	2 (2,7)	1 (1,8)	1 (5,6)	0.62
2	13 (17,8)	11 (20,0)	2 (11,1)	
3	46 (63,0)	35 (63,6)	11 (61,1)	
4	12 (16,4)	8 (14,5)	4 (22,2)	
**N**				
0	40 (54,8)	34 (61,8)	6 (33,3)	0.09
1	21 (28,8)	14 (25,5)	7 (38,9)	
2	12 (16,4)	7 (12,7)	5 (27,8)	
**M**				
0	69 (94,5)	52 (94,5)	17 (94,4)	0.99
1	4 (5,5)	3 (5,5)	1 (5,6)	
**Stage**				
I	11 (15,1)	9 (16,4)	2 (11,1)	0.24
II	28 (38,4)	24 (43,6)	4 (22,2)	
III	30 (41,1)	19 (34,5)	11 (61,1)	
IV	4 (5,5)	3 (5,5)	1 (5,6)	
**Diff grade**				
Intermed-high	58 (79,4)	48 (87,3)	10 (55,6)	0.02
Low	14 (19,2)	7 (12,7)	7 (38,9)	
*Missing*	1 (1,4)		1 (5,6)	
**Mucinous histology**				
Absent	63 (86,3)	51 (92,7)	12 (66,7)	0.01
Present	10 (13,7)	4 (7,3)	6 (33,3)	
**Vascular invasion**				
Absent	57 (78,1)	45 (81,8)	12 (66,7)	0.2
Present	16 (21,9)	10 (18,2)	6 (33,3)	
**Neural invasion**				
Absent	67 (91,8)	52 (94,5)	15 (83,3)	0.16
Present	6 (8,2)	3 (5,5)	3 (16,7)	
**Location**				
Colon	40 (54,8)	28 (50,9)	12 (66,7)	0.29
Rectum	33 (45,2)	27 (49,1)	6 (33,3)	
**Neoadj radiation (n=33)1**				
25 Gy	18 (54,5)	14 (51,8)	4 (66,7)	0.91
50,4 Gy + cap	3 (9,1)	2 (7,4)	1 (16,7)	
No	12 (36,4)	11 (40,7)	1 (16,7)	
**Adj treatment**				
Yes	19 (26,0)	8 (14,5)	11 (61,1)	<0,001
No	54 (74,0)	47 (85,5)	7 (38,9)	

^1^Rectal cancer patients only.

The present study was approved by the Ethics Committee at Lund University (ref. 210/473 and 2012/307). Written informed consent was obtained from each patient.

### Immunohistochemistry

All tumours were histopathologically re-evaluated by a board certified pathologist (KJ). For each patient, one representative paraffin block was selected from the primary tumour, and when applicable, all corresponding metastases to lymph nodes (n=32). For rectal cancer patients who underwent neoadjuvant radiation therapy, diagnostic pre-irradiation biopsies were also analyzed for PODXL expression (n=16).

For immunohistochemical analysis, full-face sections were automatically pre-treated using the PT-link system (DAKO, Glostrup, Denmark) and then stained in an Autostainer Plus (DAKO, Glostrup, Denmark) with the affinity-purified polyclonal anti-PODXL antibody HPA 2110 (Atlas Antibodies, Stockholm, Sweden, diluted 1:250). The specificity of this antibody, originally generated within the Human Protein Atlas (HPA) project, has been validated using Western blotting and protein arrays, and PODXL protein expression has been mapped by immunohistochemistry in 48 types of normal tissues and 20 common cancers (http://www.proteinatlas.org). The same antibody was used to detect PODXL expression in CRC in our previous studies [7,8] and in studies on bladder [10], testicular [12] and pancreatic [11] cancer.

### Evaluation of PODXL staining

As in previous studies, PODXL staining was recorded as negative (0), weak cytoplasmic positivity in any proportion of cells (1), moderate cytoplasmic positivity in any proportion (2), distinct membranous positivity in ≤ 50% of cells (3) and distinct membranous positivity in > 50% of cells (4) [7,8,10]. Overexpression of PODXL was considered if the tumour cells exhibited a distinct membranous staining in any proportion of the cells (3–4). Normal colorectal mucosa adjacent to the cancers functioned as negative control and tumour-associated vasculature as positive control. The staining was evaluated by two independent observers (AL and KJ) who were blinded to clinical and outcome data. Scoring differences were discussed in order to reach consensus.

### Statistics

Spearman’s Rho and Chi-square tests were used for comparison of PODXL expression and relevant clinicopathological characteristics and to analyze the concordance between PODXL expression in primary tumour and metastases and between pre- and postirradiation tumour samples. A p-value of 0.05 was considered statistically significant. All statistical analyses were performed using SPSS version 20 (SPSS Inc, Chicago, IL).

## Results

### PODXL expression in primary tumours and its association with clinicopathological parameters

Membranous PODXL expression was denoted in 18/73 (24,7%) primary tumours, Analysis of the relationship between PODXL expression in primary tumours and established clinicopathological parameters revealed a strong correlation between PODXL overexpression and low differentiation grade (p=0.020), presence of mucinous histology (p=0.010) and female gender (p<0.010). There were no statistically significant associations between PODXL expression and other clinicopathological parameters including age at diagnosis, tumour location, T-stage, N-stage and presence of vascular and neural invasion (Table 1).

### Concordance between PODXL expression in primary colorectal tumours and corresponding lymph node metastases

**Figure 1:**
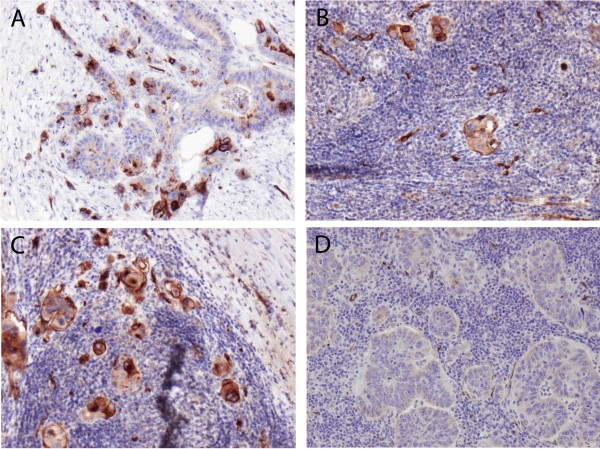
Immunohistochemical sample images from one case with positive membranous PODXL staining in the primary tumour (A), and corresponding positive (B,C) and negative (D) lymph node metastases.

PODXL expression could be evaluated in 31/32 (96,9%) patients with lymph node metastases. The level of concordance between primary colorectal tumours and related lymph node metastases was high (Table 2). In all cases with a negative primary tumour, the same status was observed in the lymph nodes. A discrepancy between positive primaries and a fraction of their corresponding lymph nodes was however noted in 7 (22,6%) cases. Sample immunohistochemical images of one discrepant case is shown in Figure 1. In one case, the primary tumour had very few positive cells and the same was seen in one of the lymph nodes, while the other two lymph nodes were negative. Considering the median value of PODXL expression in the lymph nodes for each patient, the concordance of PODXL expression between primary CRCs and lymph node metastases was 93,5% and the correlation was statistically significant (p<0.001).

**Table 2:** Concordance between PODXL expression in primary colorectal tumours and corresponding lymph node metastases

	**Tumour**	**PODXL**	**No of**	**Evaluable**	**PODXL pos**	**PODXL neg**	**Concordance**
**Patient**	**location**	**prim tumour**	**ln met**	**ln met**	**ln met (%)**	**ln met (%)**	**(%)**
1	Colon	Pos	4	4	25	75	25
2	Colon	Pos	3	3	33.3	66.7	33.3
3	Colon	Pos	20	17	58.8	41.2	58.8
4	Colon	Pos	3	3	66.7	33.3	66.7
5	Colon	Pos	5	5	80	20	80
6	Colon	Pos	6	5	80	20	80
7	Colon	Pos	2	2	100	0	100
8	Colon	Pos	2	2	100	0	100
9	Rectum	Pos	10	10	80	20	80
10	Rectum	Pos	2	2	100	0	100
11	Rectum	Pos	3	3	100	0	100
12	Colon	Neg	14	14	0	100	100
13	Colon	Neg	2	2	0	100	100
14	Colon	Neg	2	1	0	100	100
15	Colon	Neg	3	3	0	100	100
16	Colon	Neg	1	1	0	100	100
17	Colon	Neg	5	5	0	100	100
18	Colon	Neg	1	1	0	100	100
19	Colon	Neg	14	14	0	100	100
20	Colon	Neg	1	1	0	100	100
21	Rectum	Neg	4	3	0	100	100
22	Rectum	Neg	2	2	0	100	100
23	Rectum	Neg	1	1	0	100	100
24	Rectum	Neg	8	6	0	100	100
25	Rectum	Neg	1	1	0	100	100
26	Rectum	Neg	3	2	0	100	100
27	Rectum	Neg	1	1	0	100	100
28	Rectum	Neg	5	5	0	100	100
29	Rectum	Neg	2	1	0	100	100
30	Rectum	Neg	1	1	0	100	100
31	Rectum	Neg	9	9	0	100	100

### PODXL expression in rectal tumours pre- and post-irradiation

**Figure 2:**
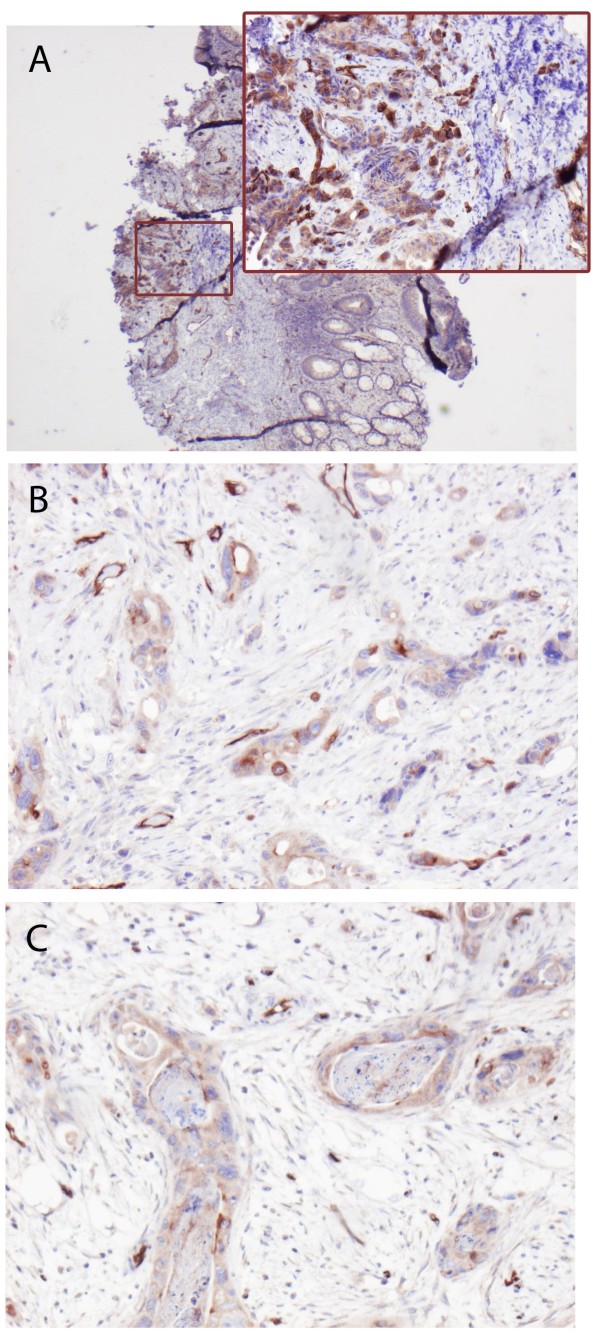
Immunohistochemical sample images from one rectal cancer case with positive membranous PODXL staining in the pre-irradiation biopsy (A), surgical resection specimen of primary tumour post-irradiation (B) and lymph node metastasis (C).

PODXL expression could be evaluated in 16/21 (76,2%) rectal biopsies pre-irradiation. Two (12,5%) were PODXL positive and 14 (87,5%) PODXL negative. A discrepancy between PODXL expression in tumours before and after radiation therapy was noted in two cases (Table 3). In both discrepant cases, positive conversion (negative in the pre-irradiation biopsy and positive in the post-irradiation tumour) was observed. Notably, 5/6 (83,3%) of the patients with PODXL positive rectal tumours in the cohort were considered high-risk patients and therefore received neoadjuvant radiation therapy. Sample immunohistochemical images are shown in Figure 2.

**Table 3:** Concordance between PODXL expression in rectal tumour samples pre- and post-irradiation therapy

	***Post-irradiation***	
***Pre-irradiation***	**PODXL neg**	**PODXL pos**
	**(n=12)**	**(n=4)**
PODXL neg	12	2
PODXL pos	0	2
		p=0,050

## Discussion

When identifying new prognostic biomarkers it is of great importance to decide which tumour site to examine, as the expression might differ in primary tumour and metastases. In this study we have analyzed PODXL expression in 31 primary tumours and a total number of 140 corresponding lymph node metastases, thus providing a thorough characterization of PODXL expression in both settings. Moreover, we have examined the potential effect of radiation therapy on PODXL expression in rectal cancer patients.

PODXL has previously been found to correlate with a poor prognosis in CRC [7,8]. So far, all results are derived from studies based on TMAs with retrospectively collected tumour samples. In this study, we used full-face sections, one from each case, to determine PODXL expression in 73 CRC patients. Our results indicate that by use of full-face section analysis, a larger proportion of tumours are identified as being PODXL positive, i.e. having membranous expression, compared to TMA-based analyses (24,7% vs 8-13%) [7,8]. These findings are not unexpected, since PODXL is, in the vast majority of cases, overexpressed in a heterogenous fashion, preferrably at the invasive tumour front. Therefore, use of the TMA-technique will most likely lead to an underestimation of positive cases. Moreover, no tumour in this cohort had membranous staining in more than 50% of the tumour cells (i.e. category 4), which may be explained by the size of the cohort, but could also be attributed to the tumour area selected for sampling. The number of cases denoted as having > 50% cells with membranous PODXL ex-pression in previous studies is however negligible, further supporting that using a cutoff based on the presence or absence of membranous staining should be sufficient for prognostication purposes. Even if recognition of membranous PODXL expression is fairly straightforward, and the mere presence rather than the quantity seems to be of prognostic importance, it would however be of interest to compare visual scoring and automated analysis in future studies [13,14].

For characterization of key molecular alterations and expression of investigative biomarkers in tumours from large patient cohorts, whether retrospectively or prospectively defined, the TMA-technology is indispensable [15]. For prospective biomarker studies and in clinical use, however, analysis of full-face sections should be the most convenient and applicable method.

Prognostic biomarkers in CRC are routinely analyzed in the primary tumor, whereas tumor cells in lymph node metastases are not characterized. Previous studies on KRAS expression have shown a discrepancy between the primary tumour and corresponding lymph node metastases [16,17], whereas the expression of other biomarkers, e.g. ER and HER-2 in breast cancer have been demonstrated to be highly concordant [18]. Of note, while most studies related to the concordance between primary and metastatic lesions have only examined a few lymph nodes (typically two per patient), we have in this study strived to examine all metastatic lymph nodes.

Although there was a discordance of PODXL expression between primary tumours and lymph node metastases in some cases, this was limited to a few cases with PODXL positive primaries where a clonal distribution of PODXL expression was observed in the metastatic lymph nodes. These results reflect the fact that the small proportion of cells in a positive primary tumour displaying membranous PODXL expression are highly prone to metastasize. The excellent concordance between primary tumour and lymph node metastases demonstrate that assessment of the primary tumour is sufficient to determine if a patient has a PODXL positive tumour. The expression of PODXL in lymph node metastases can however provide prognostic information when no primary tumour is available for analysis. Moreover, in future studies, it would be of interest to perform in-depth analyses of other molecular characteristics, and drivers of the metastatic phenotype, that may differ between PODXL negative and positive lymph node metastases in individual cases.

The significant associations between PODXL expression and several unfavourable clinicopathological characteristics (e.g. TNM stage) that have been demonstrated in our previous studies [7,8], did not reach statistical significance in this study, most likely due to the small sample size. Nevertheless, despite the small number of patients, a statistically significant relationship between PODXL expression and differentiation grade and mucinous histology was seen. Moreover, there was an overweight of PODXL positive tumours among patients who received adjuvant treatment, an indirect measurement of more aggressive tumours. The significant association of PODXL expression with female gender has not been observed in previous studies and is likely attributable to the small size of the cohort.

It is well known that approximately half of the patients with CRC stage III disease will relapse, and that adjuvant chemotherapy reduces the risk of recurrence with 20-30%. Our previous study has shown that patients with PODXL positive tumours within this group benefit from adjuvant chemotherapy irrespective of treatment regime [7]. While the majority of patients with stage III disease in this study received adjuvant treatment, a few patients were not considered candidates for chemotherapy due to old age or comorbidity. In cases of doubt whether adjuvant treatment should be given, assessment of PODXL expression may be a useful prognostic tool. Moreover, the finding of a higher proportion of PODXL positive tumours in full-face sections is of particular clinical relevance in stage II disease, where it is of uttermost importance to identify patients with high-risk disease who would benefit from adjuvant treatment. Previous studies have shown that adjuvant chemotherapy could improve survival for this group of patients, but that the incremental benefits are small [19]. Therefore, further prognostic tools are needed to better guide treatment decisions in this patient category. As PODXL expression has been demonstrated to have a prognostic value in patients with stage II disease in retrospective analysis [7] this association warrants further study in the prospective setting.

In biomarker studies it is important to consider the effect of neoadjuvant treatment on biomarker expression. In this study we found an excellent concordance between PODXL expression in rectal tumours before and after neoadjuvant irradiation, suggesting that PODXL expression is not affected by radiation therapy.

In conclusion, the results from this study suggest that PODXL expression in CRC is concordant in primary tumours and corresponding lymph node metastases in individual patients, and also remains unaffected by neoadjuvant radiation therapy. The results further support the clinical utility of PODXL as a biomarker for risk assessment in CRC, even in cases where no primary tumour is available for analysis, and irrespective of histopathological response to neoadjuvant treatment.

## Competing interests

A patent has been filed related to the use of PODXL as a prognostic biomarker in CRC.

## Authors’ contributions

AL performed statistical analysis, carried out the functional studies and drafted the manuscript. BN constructed the TMAs. IS and IP assisted with data collection. MU participated in the design of the study and provided technical assistance. JE helped with clinical advice. KJ conceived of the study, participated in its design and coordination and helped to draft the manuscript. All authors read and approved the final manuscript.

## References

[B1] Kerjaschki D Sharkey DJ Farquhar MG Identification and characterization of podocalyxin–the major sialoprotein of the renal glomerular epithelial cell J Cell Biol 1984 98 4 1591 1596 10.1083/jcb.98.4.1591 6371025 PMC2113206

[B2] Miettinen A Solin ML Reivinen J Juvonen E Vaisanen R Holthofer H Podocalyxin in rat platelets and megakaryocytes Am J Pathol 1999 154 3 813 822 10.1016/S0002-9440(10)65328-X 10079259 PMC1866405

[B3] McNagny KM Pettersson I Rossi F Flamme I Shevchenko A Mann M Graf T Thrombomucin, a novel cell surface protein that defines thrombocytes and multipotent hematopoietic progenitors J Cell Biol 1997 138 6 1395 1407 10.1083/jcb.138.6.1395 9298993 PMC2132552

[B4] Schopperle WM Kershaw DB DeWolf WC Human embryonal carcinoma tumor antigen, Gp200/GCTM-2, is podocalyxin Biochem Biophys Res Commun 2003 300 2 285 290 10.1016/S0006-291X(02)02844-9 12504081

[B5] Somasiri A Nielsen JS Makretsov N McCoy ML Prentice L Gilks CB Chia SK Gelmon KA Kershaw DB Huntsman DG Overexpression of the anti-adhesin podocalyxin is an independent predictor of breast cancer progression Cancer Res 2004 64 15 5068 5073 10.1158/0008-5472.CAN-04-0240 15289306

[B6] Casey G Neville PJ Liu X Plummer SJ Cicek MS Krumroy LM Curran AP McGreevy MR Catalona WJ Klein EA Podocalyxin variants and risk of prostate cancer and tumor aggressiveness Hum Mol Genet 2006 15 5 735 741 10.1093/hmg/ddi487 16434482

[B7] Larsson A Johansson ME Wangefjord S Gaber A Nodin B Kucharzewska P Welinder C Belting M Eberhard J Johnsson A Overexpression of podocalyxin-like protein is an independent factor of poor prognosis in colorectal cancer Br J Cancer 2011 105 5 666 672 10.1038/bjc.2011.295 21829192 PMC3188928

[B8] Larsson A Fridberg M Gaber A Nodin B Leveen P Jonsson G Uhlen M Birgisson H Jirstrom K Validation of podocalyxin-like protein as a biomarker of poor prognosis in colorectal cancer BMC Cancer 2012 12 282 10.1186/1471-2407-12-282 22769594 PMC3492217

[B9] Cipollone JA Graves ML Kobel M Kalloger SE Poon T Gilks CB McNagny KM Roskelley CD The anti-adhesive mucin podocalyxin may help initiate the transperitoneal metastasis of high grade serous ovarian carcinoma Clin Exp Metastasis 2012 29 3 239 252 10.1007/s10585-011-9446-0 22262060

[B10] Boman K Larsson AH Segersten U Kuteeva E Johannesson H Nodin B Eberhard J Uhlen M Malmstrom PU Jirstrom K Membranous expression of podocalyxin-like protein is an independent factor of poor prognosis in urothelial bladder cancer Br J Cancer 2013 108 11 2321 2328 10.1038/bjc.2013.215 23652315 PMC3681027

[B11] Dallas MR Chen SH Streppel MM Sharma S Maitra A Konstantopoulos K Sialofucosylated podocalyxin is a functional E- and L-selectin ligand expressed by metastatic pancreatic cancer cells Am J Physiol Cell Physiol 2012 303 6 C616 C624 10.1152/ajpcell.00149.2012 22814396 PMC3468350

[B12] Cheung HH Davis AJ Lee TL Pang AL Nagrani S Rennert OM Chan WY Methylation of an intronic region regulates miR-199a in testicular tumor malignancy Oncogene 2011 30 31 3404 3415 10.1038/onc.2011.60 21383689 PMC3117973

[B13] Laurinaviciene A Dasevicius D Ostapenko V Jarmalaite S Lazutka J Laurinavicius A Membrane connectivity estimated by digital image analysis of HER2 immunohistochemistry is concordant with visual scoring and fluorescence in situ hybridization results: algorithm evaluation on breast cancer tissue microarrays Diagn Pathol 2011 6 87 10.1186/1746-1596-6-87 21943197 PMC3191356

[B14] Rizzardi AE Johnson AT Vogel RI Pambuccian SE Henriksen J Skubitz AP Metzger GJ Schmechel SC Quantitative comparison of immunohistochemical staining measured by digital image analysis versus pathologist visual scoring Diagn Pathol 2012 7 42 10.1186/1746-1596-7-42 22515559 PMC3379953

[B15] Torhorst J Bucher C Kononen J Haas P Zuber M Kochli OR Mross F Dieterich H Moch H Mihatsch M Tissue microarrays for rapid linking of molecular changes to clinical endpoints Am J Pathol 2001 159 6 2249 2256 10.1016/S0002-9440(10)63075-1 11733374 PMC1850582

[B16] Han CB Li F Ma JT Zou HW Concordant KRAS mutations in primary and metastatic colorectal cancer tissue specimens: a meta-analysis and systematic review Cancer Invest 2012 30 10 741 747 10.3109/07357907.2012.732159 23075074

[B17] Miranda C Nucifora M Molinari F Conca E Anania MC Bordoni A Saletti P Mazzucchelli L Pilotti S Pierotti MA KRAS and BRAF mutations predict primary resistance to imatinib in gastrointestinal stromal tumors Clinical cancer research: an official journal of the American Association for Cancer Research 2012 18 6 1769 1776 10.1158/1078-0432.CCR-11-2230 22282465

[B18] Falck AK Ferno M Bendahl PO Ryden L Does analysis of biomarkers in tumor cells in lymph node metastases give additional prognostic information in primary breast cancer? World J Surg 2010 34 7 1434 1441 10.1007/s00268-010-0499-z 20213203

[B19] Gray R Barnwell J McConkey C Hills RK Williams NS Kerr DJ Adjuvant chemotherapy versus observation in patients with colorectal cancer: a randomised study Lancet 2007 370 9604 2020 2029 10.1016/S0140-6736(07)61866-2 18083404

